# Unraveling the Mechanism of Action of Myricetin in the Inhibition of hUba1∼Ubiquitin Thioester Bond Formation via *In Silico* Molecular Modeling Techniques

**DOI:** 10.1021/acsomega.3c03605

**Published:** 2023-08-08

**Authors:** Paras Gaur, Chetna Tyagi

**Affiliations:** † Institute of Genetics, Biological Research Centre, Temesvári krt. 62, 6726 Szeged, Hungary; ‡ Department of Microbiology, Faculty of Science and Informatics, University of Szeged, Közép fasor 52, H-6726 Szeged, Hungary

## Abstract

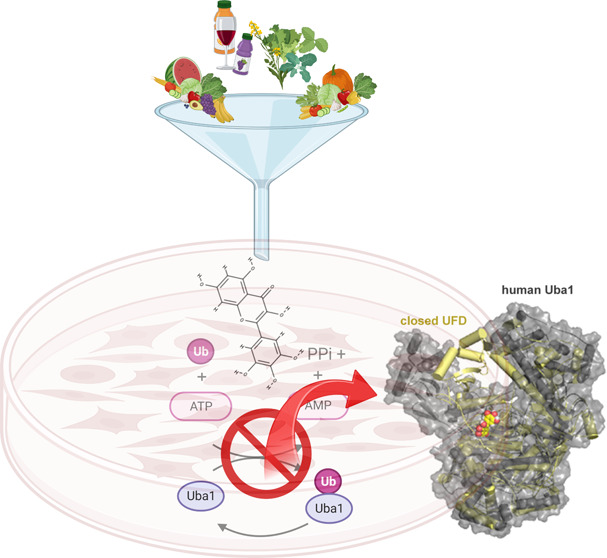

Ubiquitination is a crucial type of protein modification which helps to control substrate degradation and maintain cell homeostasis. Recent studies suggest that ubiquitination and deubiquitination are involved in regulating metabolic reprogramming in cancer cells and maintaining cancer stem cells. Uba1, a crucial protein in the ubiquitination cascade, can be targeted to develop effective inhibitors for cancer treatment. In previous work, we showed that myricetin (Myr) acts as a potential human Uba1 (hUba1) inhibitor. In this study, we have utilized computational modeling techniques to attempt to illustrate the mechanism of action of Myr. Through extra-precision docking, we confirmed that Myr binds to the adenosine triphosphate (ATP)-binding site of hUba1 (referred to as hotspot 1) with the highest binding affinity. The dynamics of this interaction revealed that hUba1 undergoes a conformational shift from open to closed upon binding of Myr. Myr also migrates outward to interact with the crossover loop simultaneously as the rotational shift of the ubiquitin fold domain (UFD) takes place, thereby blocking access to the ubiquitin binding interface of hUba1 and the crossover loop. The outward migration also explains the reversible nature of Myr binding to hUba1 in previous experiments. We hypothesize that Myr acts as an inhibitor of Uba1∼Ub thioester bond formation by causing a large domain shift toward a closed conformation. Few other analogues of Myr containing the same flavone skeleton showed promising docking scores against hUba1 and could be considered for further validation. We propose that Myr and some of its analogues reported in this study may be promising candidates for developing effective Uba1 inhibitors for cancer treatment.

## 1 Introduction

The discovery of the field of ubiquitination can be traced back to 1977 when a study on histones reported a DNA-associated protein with a unique structure, consisting of one C-terminus and two N-termini. The protein had a Y-shape with its short arm joined to an internal lysine of histone H2A through its C-terminus. This short arm was later identified as ubiquitin by L. Hunt and M. Dayhoff. Over the past decade, numerous studies have been conducted that have uncovered the link between ubiquitination and cancer.^1−3^ Ubiquitination involves the covalent attachment of ubiquitin, a small and evolutionarily conserved protein, to a lysine residue on a substrate protein via an isopeptide bond. This modification can serve various purposes including preparing the tagged protein for proteasomal degradation, as well as nonproteolytic functions such as facilitating multiprotein complex assembly, regulating enzymatic activity, modulating DNA repair, and initiating inflammatory signaling or autophagy.^4−6^ While lysine residues are the most common sites for ubiquitination, other amino acids such as cysteine, serine, and threonine residues, as well as the N-terminal amino group of proteins, can also be targeted for ubiquitination.^7^

Ubiquitination can be classified into two categories: monoubiquitination and polyubiquitination. Monoubiquitination refers to the attachment of a single ubiquitin moiety to a lysine residue of the target protein, while polyubiquitination involves the successive ubiquitination of the target protein with additional ubiquitin moieties, resulting in the formation of a ubiquitin chain.^8,9^ The classical process of ubiquitination involves three steps.^10^ First, the E1 ubiquitin-activating enzyme (Uba1; also known as UBE1) activates ubiquitin by forming a high-energy thioester bond between the C-terminus of ubiquitin and the active-site cysteine on UBE1, which requires adenosine triphosphate (ATP) hydrolysis and results in the formation of a charged ubiquitin-AMP intermediate. Second, the activated ubiquitin is transferred to the catalytic cysteine on an E2 ubiquitin-conjugating enzyme. And lastly, the E3 ubiquitin ligase binds to both the E2-ubiquitin complex and the substrate protein, facilitating the transfer of ubiquitin from the E2 to a specific lysine residue on the substrate protein for ubiquitination.^4−6,11^ The dysregulation of ubiquitination is a crucial factor in the development of various diseases including cancer. Cancer cells exhibit malignant properties and alter metabolic activities to support their growth and adapt to stressful conditions. Ubiquitination plays an important role in controlling substrate degradation and maintaining cell homeostasis. Recent studies have observed that the process of ubiquitination participates in the modulation of cancer metabolism, with the ubiquitination of key proteins such as RagA, mTOR, PTEN, AKT, cMyc, and p53 significantly regulating the activity of various signaling pathways. The ubiquitination of core stem cell regulators and members of the Wnt and Hippo-YAP signaling pathways also participates in the maintenance of cancer stem cell stemness.^12−14^ Understandably, ubiquitination has become a preferred target for drug identification against cancer. Recently, Haracska lab reported a high-throughput screening of thousands of molecules using Alpha-based ubiquitination assays, through which they discovered a few molecules that target the E1:human Uba1 protein in the ubiquitination cascade and E2-E3 (Rad6-Rad18).^10,15−17^ They confirmed that the green tea polyphenol (−)-epigallocatechin-3-gallate (EGCG), (−)-epicatechin-3-gallate (ECG), and (−)-epigallocatechin (EGC) and Myr specifically targeted the Uba1 protein. Myr and EGCG were found to be particularly effective as they exhibited inhibitory activity for both the Uba1-Ub thioester assay and PCNA ubiquitination.^10,15,16,18^ Following this, we published a molecular modeling study to understand the mode of action of EGCG and its derivatives with hUba1 as the target.^16^

In this study, we would like to report the plausible mechanism of action of Myr as its experimental activity was vastly different from that of EGCG. Myr is a flavonoid that is commonly found in the form of glycosides in various natural fruits and flowers, including blueberry leaves, rose petals, oranges, sea buckthorn, chia seeds, and others.^19^ Recent studies have revealed that it plays diverse roles in biological processes, including but not limited to exhibiting anti-inflammatory, anticancer, antibacterial, and antiviral effects, protecting against neurological damage and obesity, as well as safeguarding against injuries.^20−27^ It is a lipophilic and weak acidic compound that works best at pH 2.0 and with low aqueous solubility (16.60 g/mL), making it insoluble in the gastrointestinal tract and thus limiting its efficacy via oral absorption.^28−30^ The target Uba1 is a multidomain protein that initiates the process of ubiquitination. The crystal structure of hUba1 protein (PDB ID: 6dc6) consists of various domains including the inactive adenylation domain (IAD) and active adenylation domain (AAD) which forms a pseudo-dimeric adenylation domain that acts as a rigid body for the overall structure. The first catalytic cysteine domain (FCCH) is connected to AAD via the β7 and β14 loops, while the second catalytic cysteine domain (SCCH) is connected to the AAD through the crossover and re-entry loops. The ubiquitin-fold domain (UFD) is connected to the AAD by a crossover loop that plays an important role in providing the interface for ubiquitin binding and its stability. The domain organization of Uba1 produces a Y-shaped structure where the pseudo-dimeric adenylation domain forms the enzyme’s base. Positioned at the top of the enzyme and facing each other are the SCCH and UFD domains, with a significant space between them that allows for the E2 ubiquitin-conjugating enzyme to fit in during the E1–E2 ubiquitin thioester transfer step in ubiquitination cascades.^31−35^

The FTMap analysis of the Uba1 structure has identified four hot spot (HS) pockets that could serve as potential sites for binding of small-molecule inhibitors. The highest scoring pocket is HS-1, which corresponds to the ATP-binding site. HS-2 is also promising and is located between the UFD and AAD domains where E2 proteins bind during the trans-thioesterification reaction. HS-3 is formed by residues from several α-helices on the SCCH domain, while HS-4 is defined by residues from the β5 strand, H7, the β4–H5 loop, and the H7–H8 loop at the bottom of the IAD. These findings suggest that targeting these HS pockets could lead to the development of effective Uba1 inhibitors.^35^ In this study, we employed a computational docking approach to investigate the possible binding mechanism of Myr and its analogues to the Uba1 protein by sampling docking poses at the three top scoring hotspots. The fourth one was not considered further due to weak binding affinity in preliminary docking trials. The best scored docking pose was then simulated using an enhanced sampling technique, and the results have been used to outline a plausible mechanism of action of Myr against hUba1.

## 2 Results and Discussion

### 2.1 Mode of Binding of the Myr to hUba1 at the Four Binding Sites (Hotspots)

The highest docking score for Myr was observed for HS-1, also the ATP-binding site, similarly to TAK-243, which is an adenosyl sulfamate that inhibits UBA1 by binding to the ATP-binding site from where it attacks the thioester-bound ubiquitin so that a ubiquitin∼TAK-243 adduct is formed that cannot be released, which blocks further ubiquitin activation.^36^ The docking score and relative extra precision (XP) scores are listed in Table 1.

**Table 1:** Binding Scores in kcal/mol for Myr Binding to the Three Hotspots of Uba1

(in kcal/mol)	HS-1	HS-2	HS-3
docking score	–11.46	–7.71	–7.36
XP score	–11.49	–7.75	–7.36
SP score	–7.02	–6.06	–5.82

**Figure 1:**
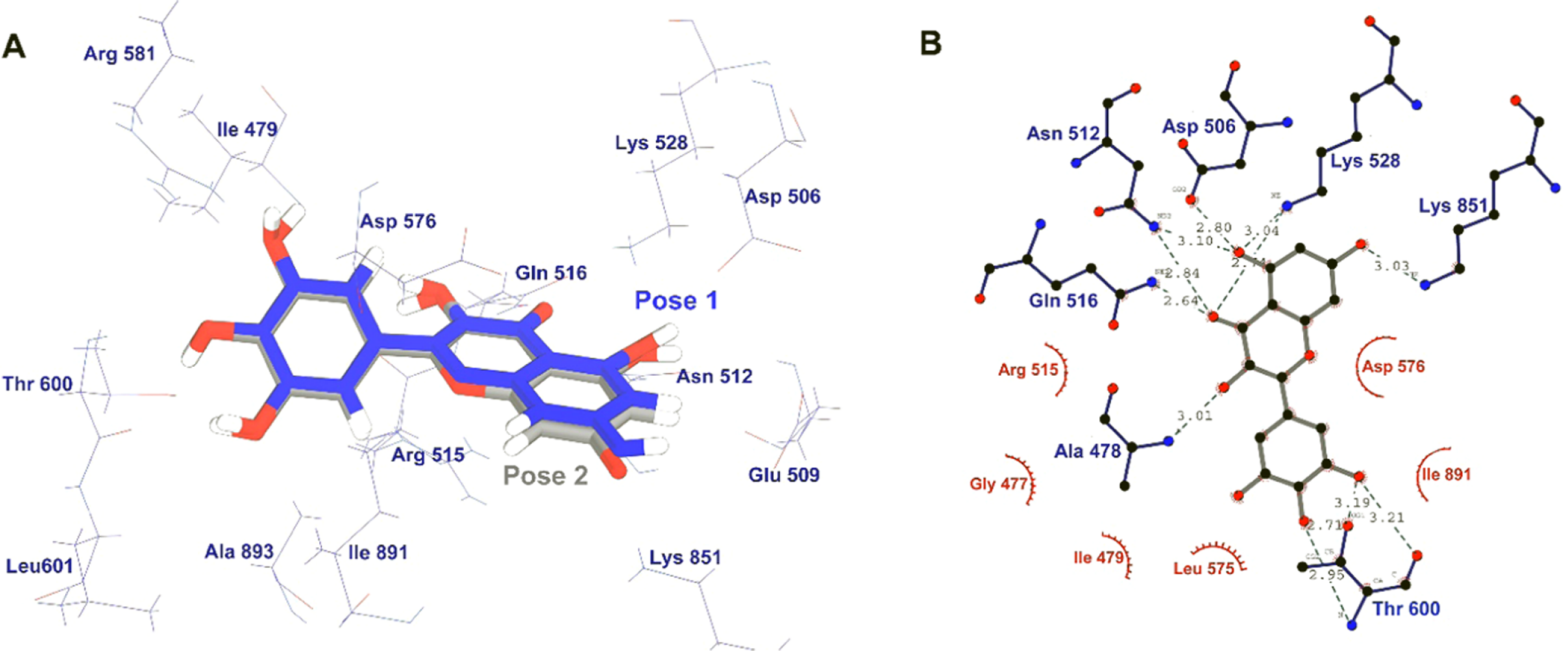
(A) Top two binding poses of Myr at HS-1 where blue is Pose 1 and gray is Pose 2, (B) binding interactions of Myr at HS-1 involving several hydrogen bonds (blue residues) and hydrophobic interactions (red residues).

An XP docking run resulted in two highly plausible binding poses with hUba1 ranked using the glide “*emodel*” scoring method. The two poses show different XP *glidescore* values, which essentially approximate the ligand binding free energy, −11.49 kcal/mol for Pose 1 and −9.32 kcal/mol for Pose 2. Upon superimposition of the two poses at the hotspot 1 site of hUBa1 (Figure 1a), it looks almost the same, and yet, the *emodel* score was found to be higher for the first pose with a value of −73.28 kcal/mol while Pose 2 was scored at a value of −69.85 kcal/mol. The important residues involved in the binding are Ala478, Asp506, Asn512, Gln516, Lys528, and Lys581 forming hydrogen bonds with the oxygen atoms of Myr (Figure 1b). Lv et al. reported the important residues involved in ATP binding in the case of hUba1.^35^ The γ-phosphate of ATP engages with Arg57, Arg515, and **Lys851**, while the β-phosphate of ATP engages in contact with **Asp506**, **Asn512**, **Lys528**, and **Asp576**. Upon docking, they found that residues Ala478 and Asp576 seem to be crucial for engaging α-phosphate of ATP, its ribose group may show interaction with Gly475 and Asp504, and its adenine group may show interaction with Arg551, Val552, **Leu575**, and Asn577. Other previous studies also highlighted the importance of **Asp576** and **Lys528** for substrate binding, preferably ATP, and for stabilization of Uba1-catalyzed ubiquitin adenylation. Upon comparison with Myr binding, it is clear that residues **Asp506**, **Asn512**, **Lys528**, and **Asp576**, which engage with β-phosphate of ATP, are crucial for the stability of the complex.

Moreover, after a comparison with the ubiquitin binding interface, it was found that Myr interacts with crucial residues of hUba1 implicated in interacting with ubiquitin. For example, it is known that Gly75 of ubiquitin forms hydrogen bonds to both the Arg581 sidechain and with Thr600 of the hUba1 AAD domain. The Gly75 of ubiquitin also forms van der Waals contacts with the Ile891 sidechain belonging to the SCCH domain of hUba1, ensuring its stability. Three H-bonds were observed with the catechol moiety of Myr (B ring) involving the Thr600 sidechain, while Ile891 was found in a hydrophobic interaction with Myr.

In previous work which involved cathechin gallates, alkyl gallates, and Myr, it was shown that Myr inhibits Uba1∼ubiquitin thioester formation when preincubated with Uba1 before adding ubiquitin and also when preincubated with ubiquitin first, unlike EGCG which only shows inhibition when preincubated with Uba1 before adding ubiquitin.^15,16^ Therefore, it is plausible that Myr does not follow in the footsteps of TAK-243 and does not form an irreversible covalent ubiquitin∼Myr adduct.^37^ The authors of the previous study, however, noted that the strong activity of Myr can be attributed to its Michael acceptor (α, β-unsaturated carbonyl) functionality, a potentially reactive electrophile susceptible to attack by nucleophilic groups on proteins. However, they found that Myr, like EGCG, inhibits ubiquitination in a reversible manner which is not characteristic of a covalent Michael addition, even though the authors indicated a slight possibility.^15,16^

A study by Niu et al.^38^ reported the plausible mode of binding of Myr with the Suilysin monomeric virulence factor of Gram-positive bacteria *Streptococcus suis*. Molecular dynamics (MD) simulations and principal component analysis (PCA) revealed that binding of Myr at the gap regions between domains 2 and 3 restricts the conformational transition to oligomeric forms.^38^ Another study by Song and Shao, 2022, described the mode of binding of Myr with ectonucleotide pyrophosphatase/phosphodiesterase 1 (ENPP1), a protein crucial for immune cell penetration into tumors. Based on molecular docking, a pi–pi interaction (ring stacking) involving the benzopyrone scaffold (2-phenyl group) of Myr and rings of Phe257 and Tyr340 residues of ENPP1 was reported.^39^ We also checked for a similar interaction that may stabilize Myr binding with Uba1; however, no such ring sidechain containing amino acid residues were observed in our docking poses.

**Figure 2:**
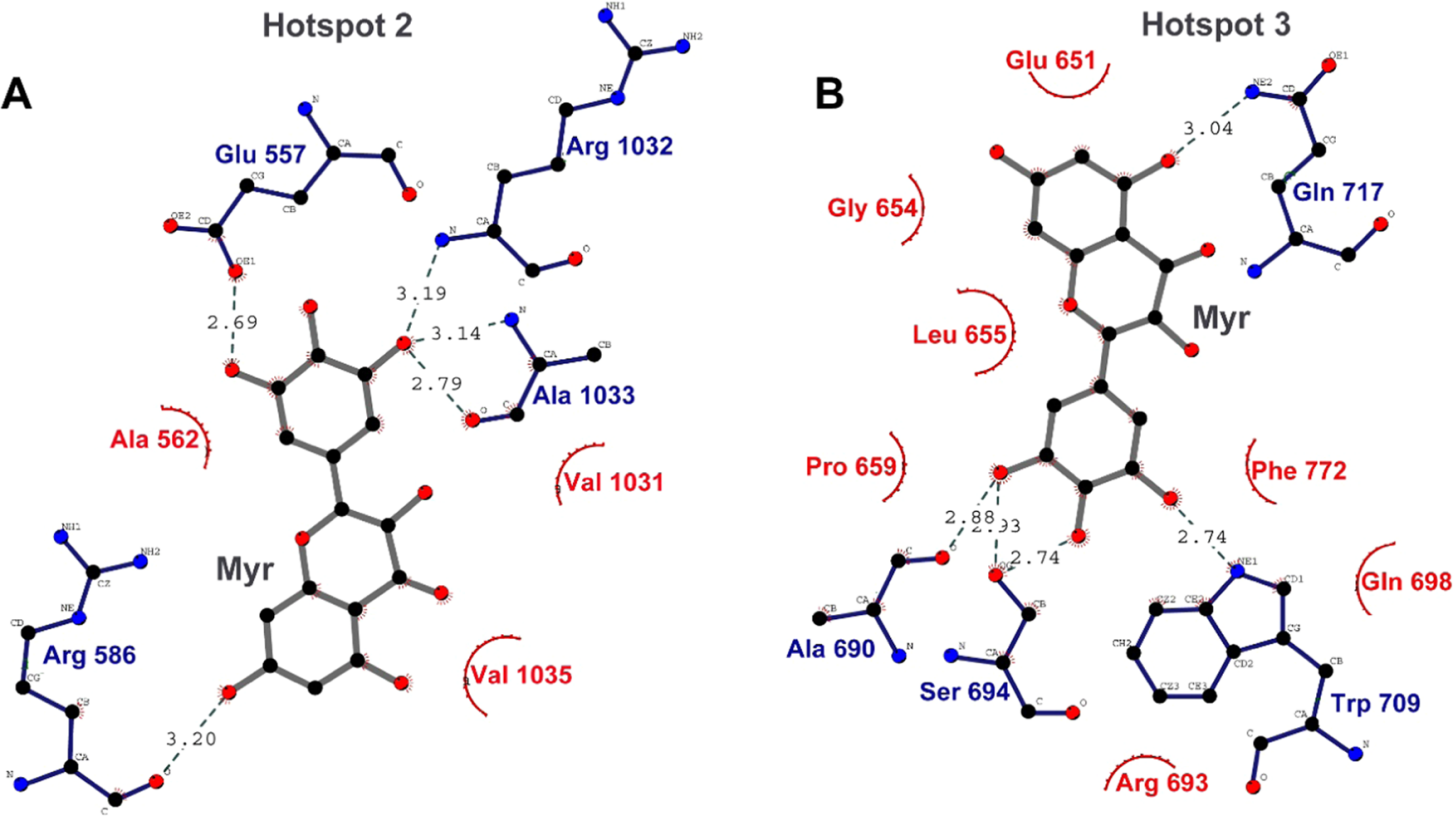
Binding poses of Myr at (A) HS-2 and at (B) HS-3. The hydrogen bonds are marked with blue residues and hydrophobic interactions with red residues.

Apart from the highest docking pose at HS-1, the other two hotspots were also checked for docking poses with Myr. HS-2 is located between the UFD and AAD which is in proximity to where E2 proteins bind during the trans-thioesterification reaction. HS-3 is formed by residues from α-helices H19, H20, H22, H23, and H25 on the SCCH domain. Figure 2 describes the binding interactions of Myr at HS-2 (Figure 2a) and HS-3 (Figure 2b). As listed in Table 1, the scores are significantly lower than at HS-1, which indicates, without doubt, that the binding site of Myr on hUba1 is at the ATP-binding site.

### 2.2 Multiple Analogues of Myr Chosen for Evaluation of Binding Affinity with hUba1

Many known structural analogues of Myr (a hexahydroxyflavone), sharing the base flavone structure, were selected for docking studies targeting hUba1. These other naturally occurring flavonoids are apigenin, diosmetin, dihydromyricetin, fisetin, hesperetin, isoquercetin, kaempferol, luteolin, morin, quercetin, tangeretin, and taxifolin. The docking scores (in kcal/mol) of each of these analogues against the four top scoring hotspot sites are summarized in Table 2. Specifically, at HS-1, where Myr shows the highest docking score (more negative values), fisetin, isoquercetin, kaempferol, morin, and quercetin resulted in the highest docking scores (below −7.00 kcal/mol) and are highlighted in bold. Others like dihydromyricetin, isoquercetin, and quercetin also showed comparably higher docking scores at other hotspots than at HS-1 and may indicate toward a different mechanism of action than Myr. The analogues show weaker binding affinity to hUba1 in comparison to Myr, and yet, we decided to report it in this work as it is an important result for future work. The experiments provide a clue into the structural qualities of various functional groups attached to the flavone moiety. Whether these groups enhance or decrease the overall activity is an important information for future design of effective hUba1 inhibitors.

**Table 2:**
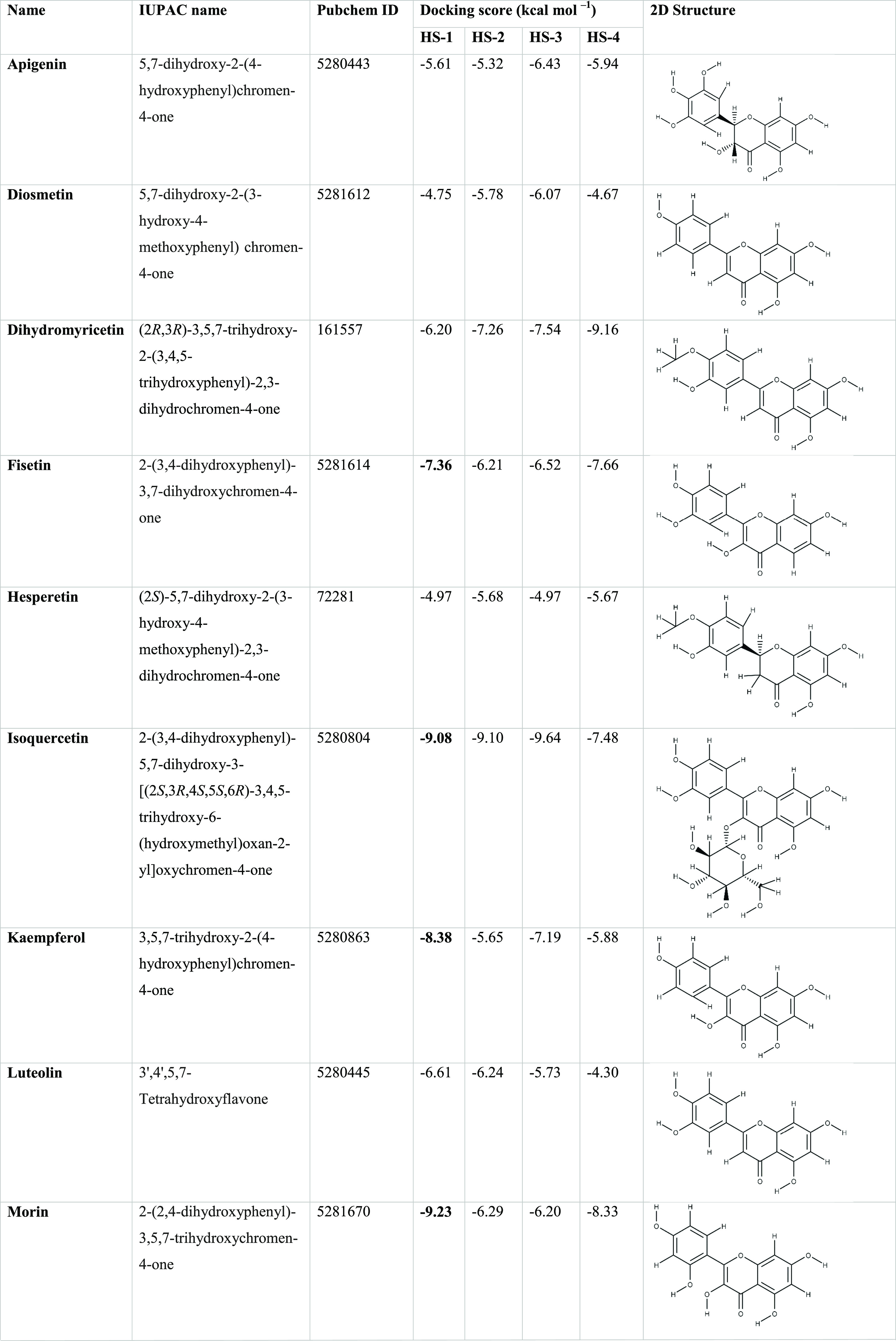

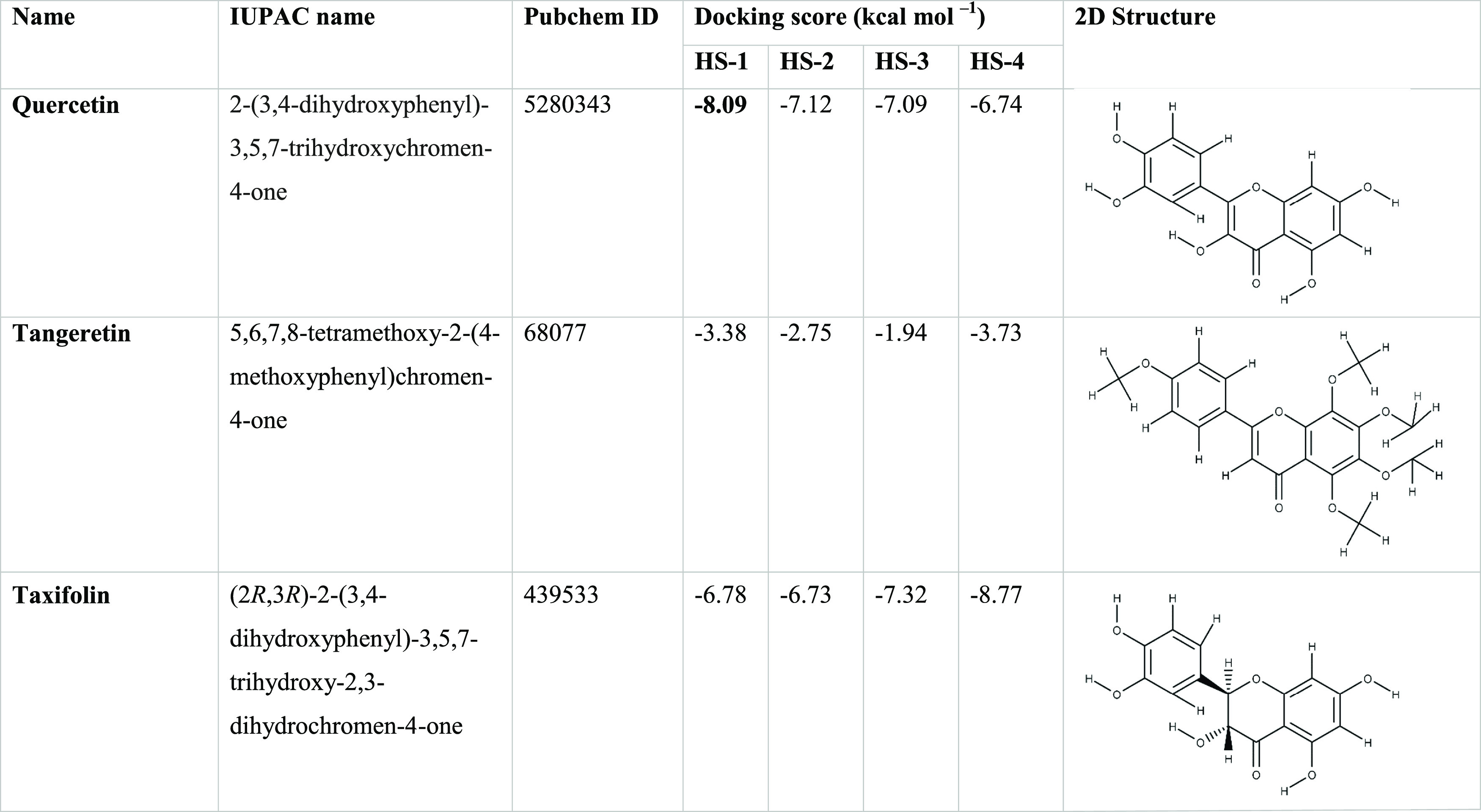
Analogues of Myr Tested for Binding Scores against hUba1; Their IPUAC Names, Pubchem IDs, and the Two-dimensional (2D) Structures^a^

^a^ The compounds with docking scores at HS-1 lower than −7.00 kcal/mol (more negative values means higher binding affinity with the protein) are highlighted in bold.

### 2.3 ADME of Top Scoring Compounds

Based on high docking scores at the HS-1 binding site, five analogues of Myr, namely, fisetin, isoquercetin, kaempferol, morin, and quercetin, were analyzed for their ADME (ADME stands for absorption, distribution, metabolism, and excretion) properties and compared against those of Myr. These compounds exhibit varying degrees of oral bioavailability and distribution throughout the body. They undergo metabolism primarily through phase II conjugation reactions and are eliminated mainly via urine and feces. The ADME properties can vary depending on factors such as formulation and dose and play a crucial role in determining the pharmacokinetic and pharmacodynamic profiles of the compounds.^40,41^ The SWISS-ADME calculates a bioavailability radar for a concise display of drug-likeness based on six physicochemical properties: lipophilicity, size, polarity, solubility, flexibility, and saturation. The optimal range for each property is represented by the pink area in the radar; **lipophilicity**: **XLOGP3** between −0.7 and +5.0, **size**: MW between 150 and 500 g/mol, **polarity**: topological polar surface area (**TPSA**) between 20 and 130 Å^2^, **solubility**: log *S* not higher than 6, **saturation**: fraction of carbons in the sp^3^ hybridization not less than 0.25, and **flexibility**: no more than nine rotatable bonds. The radar plots for all selected compounds are provided in Supporting Information File S2.

In the case of Myr, it was predicted to be too unsaturated and too polar and, therefore, not an ideal drug-likeness indicator. For saturation, the ratio of sp^3^ hybridized carbons over the total carbon count of the molecule (fraction Csp3) should be at least 0.25. Moreover, a prediction of drug-likeness according to the Lipinski (The Lipinski rule-of-five), Ghose, Veber, Egan, and Muegge methods resulted in negative feedback from Veber, Egan, and Muegge methods due to high TPSA values while a positive drug-likeness from Lipinski and Ghose methods. In comparison, fisetin, kaempferol, morin, and quercetin with positive feedback for drug-likeness from all five methods and polarity within the accepted range seem to be better candidates as lead compounds than Myr. On the contrary, isoquercetin resulted in a saturation parameter within the range but a too high polarity value, making it less drug-like and scored a negative evaluation on all five scales.

Cytochrome P450 (CYP) are commonly studied for their pharmacokinetic value in metabolism of xenobiotics. The alteration in CYP levels by a plausible drug candidate may lead to an increase/decrease in concentration of that drug in the plasma. The SwissADME analysis predicted the interaction of Myr against other CYP1A2 and CYP3A4 as an inhibitor while for CYP2C19, CYP2C9 and CYP2D6 as a noninhibitor. Bhatt et al. have recently reported the inhibition potential of Myr against **CYP2C8**, which is crucial in drug pharmacokinetics, using *in silico*, *in vitro*, and *in vivo* experiments.^42^ This analysis was carried out to understand the druggability of Myr and its analogues and aid the other *in vitro* and *in silico* results showing its promising therapeutic value as an anticancer drug.

### 2.4 Enhanced Sampling-Based Molecular Dynamics Simulations Reveal Major Conformational Shift in hUba1

**Figure 3:**
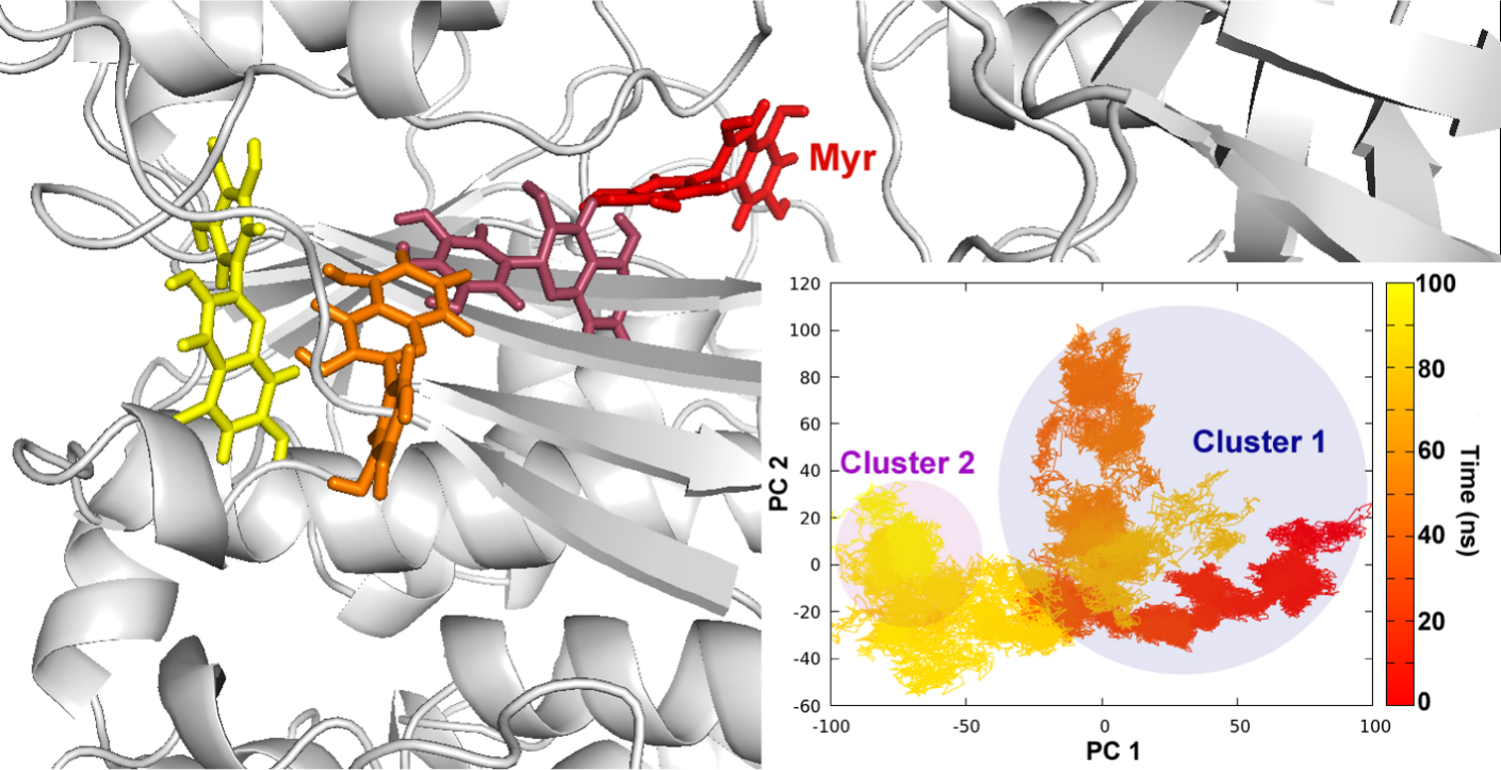
Migration of Myr from the HS-1 (in red) toward the crossover loop (in yellow) of hUba1. The inset figure plots the first two principal components which describe the movement of Myr throughout the simulation. Clustering based on structural RMSD revealed two main clusters of Myr poses.

hUba1 bound with Myr at HS-1 was simulated for a time length of 100 ns using an enhanced sampling technique known as accelerated MD (aMD) simulations to reveal the conformational changes that take place due to ligand binding, if any. The simulations revealed two major states of hUba1, the one with Myr bound to HS-1 and the second in which the SCCH domain rotates to close the Y-shaped gap and Myr moves out of the ATP-binding site (HS-1). Principal component analysis (PCA) carried out on the movement of Myr in the coordinate space can be visualized through Figure 3, where Myr (red) can be seen at the original binding pose (snapshot at 20 ns) and gradually move toward the outer crossover loop (Myr shown in yellow at 90 ns).

**Figure 4:**
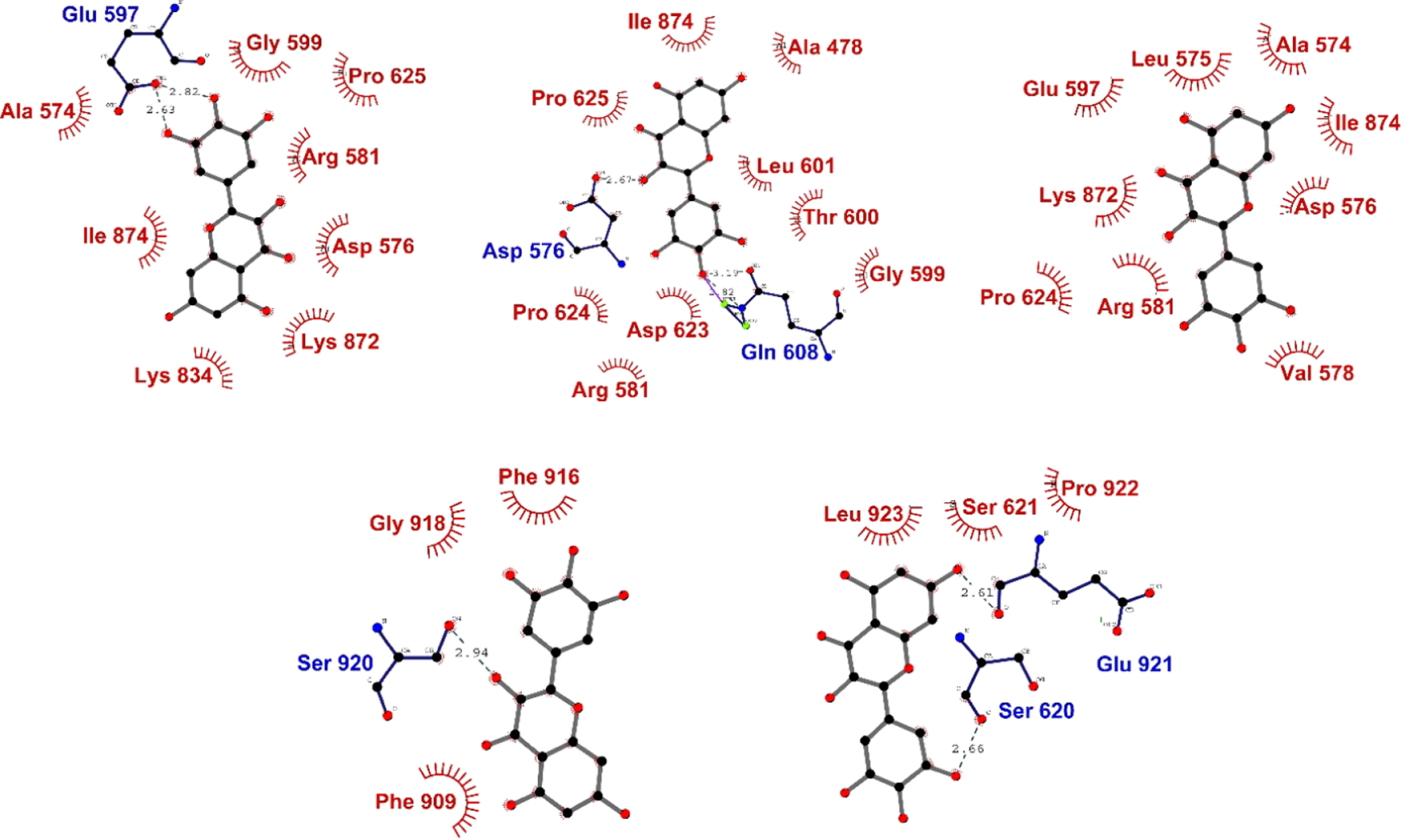
Screenshots of evolving interaction between Myr and hUba1 taken at every 20 ns snapshot of the simulation trajectory.

The PC trajectory plotted in the inset figure (Figure 3) follows the same color scheme with red → yellow migration of Myr from the AAD to crossover loop. The first cluster of conformations encompasses the majority of the simulation, but the last 20 ns represent the second cluster where Myr interacts with the crossover loop of hUba1. To understand the nature of this movement, we calculated the protein–ligand interactions for snapshots taken at every 20 ns where the dynamics of ligand interaction could be defined (Figure 4).

Lv et al. described the hUba1∼Ub interaction based on three interfaces, where interface 3 involves residues from the AAD and crossover loop of hUba1.^35^ The AAD site forms our HS-1 in this study, and therefore, we are interested if Myr binding may affect the interaction with ubiquitin. The crossover loop connects the catalytic cysteine domain to the adenylation domain and plays a crucial role in bringing these two active sites into close spatial proximity. As per Lv et al.,^35^ interface 3 likely guides the C-terminus of Ub (Arg72) toward the hUba1 active site, specifically interactions with Gln608 of the AAD and **Ser621** and Asp623 of the crossover loop. Arg72 of Ub also interacts with Asn606 of the AAD and Tyr618 of the crossover loop of hUba1. Similarly, Leu73 of ubiquitin engages with Leu601, Lys604, Gly605, and Asn606 of the AAD and Arg74 of Ub engages with Arg581 (AAD) and Glu626 (crossover loop). The crossover loop plays an important role in stabilizing the interaction with ubiquitin; for example, Ser621 and Asp623 in the crossover loop of hUba1 engage with Arg42 from ubiquitin, and Gln622 and Pro624 engage with Asp39 and Gln40 of ubiquitin.

**Figure 5:**
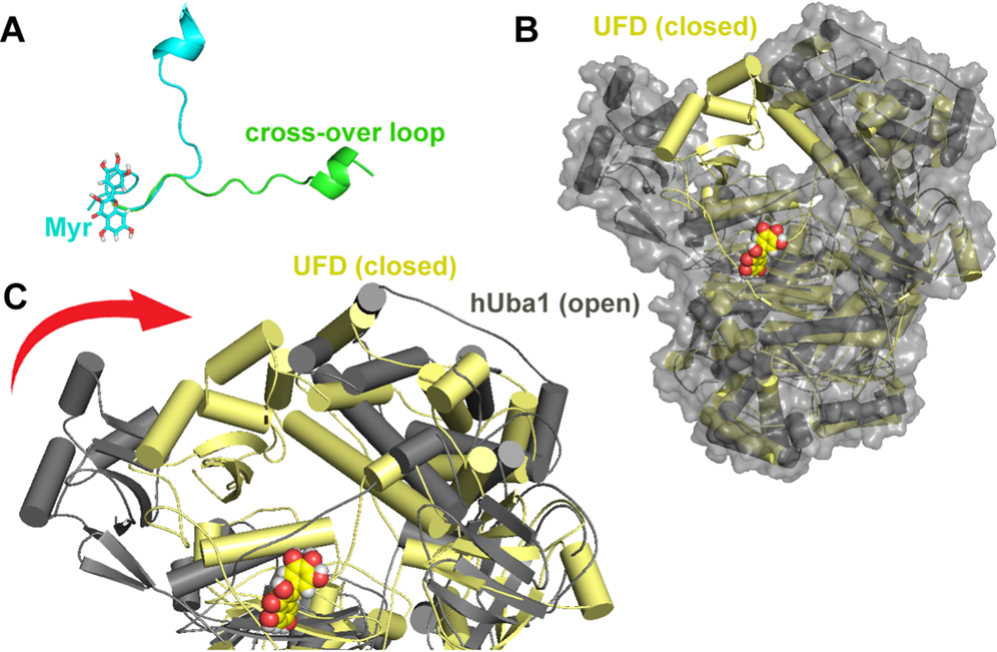
Major conformational shifts observed in the hUba1 structure along the simulation timeline in response to Myr binding. (A) Superimposition of the crossover loop region from the two conformations of hUba1 shows an ∼90° shift from open (green) to closed (cyan) forms, (B) full structure representation of hUba1 in open (gray surface style) and closed (yellow ribbon style) to show the rotation of the UFD domain, and (c) rotation of the UFD domain shown by superimposition of the open and closed conformations.

The engagement of Myr with the crossover loop toward the end of the simulation may be an indication of its mechanism of action in inhibiting hUba1∼Ub thioester bond formation. The Mg-ATP bound state of Uba1 is defined as the open state which is achieved as the crossover loop rotates to bring the catalytic cysteine closer to the adenylation site. The superimposition of the Mg-ATP bound open state with Myr at HS-1 (beginning of the simulation) to the closed state of hUba1 with Myr at the crossover loop (last 20 ns of simulation) revealed a clear rotation of the crossover loop (Figure 5).

The open and closed conformational forms of Uba1 have also been studied from the view of UFD domain motion where its proximity to the SCCH domain is proximal/closed and its remoteness is distal/open conformation. All Uba1 structures reported using experimental techniques fall between these two extremes. The original hUBa1 structure taken for this study (PDB: 6dc6) is in the Ub bound form, and therefore, UFD was in the distal conformation that widens the canyon.^35^ The superimposition of the two structures hUba1 with Myr at HS-1 (beginning of the simulation) to the closed state of hUba1 with Myr at the crossover loop (last 20 ns of simulation) shows a clear rotation of the UFD domain from distal to proximal conformation (Figure 5). Therefore, keeping in view of simulation results, we hypothesize that Myr originally targets the ATP binding site (HS-1) and then migrates outward toward the crossover loop which causes a rotational shift in the loop and, in turn, the SCCH domain, which eventually closes the Y-shaped gap. This domain shift observed during aMD simulations can be visualized in the Supporting Information video file (Supporting Information File S1). Consequently, the interface which interacts with ubiquitin is blocked and hUba1∼Ub thioester bond formation is stalled.

## 3 Conclusions

In this study, we focused on investigating the effects of binding of Myr to human Uba1 protein, a ubiquitin activation enzyme, leading in the process of ubiquitination and implicated in multiple cancer progression processes. A previous study has reported experimental proof of strong inhibition of hUba1 by Myr. Therefore, we attempted to explore the actual binding mechanism underlying this interaction using advanced computational techniques. The results from docking experiments and aMD simulations suggest that Myr undoubtedly binds to the ATP binding site (HS-1 in this work) and subsequently migrates outward toward the crossover loop which connects the AAD domain to the SCCH and UFD domains. This migration causes an ∼90° shift in the crossover loop and the rotation of the UFD domain leading to the closure of the Y-shaped gap. We hypothesize that due to this domain rotation, the interface responsible for interacting with ubiquitin is obstructed. Moreover, the crossover loop, which is crucial for stabilizing hUba1∼Ub interactions, may also become unavailable after interaction with Myr. Therefore, these conformational modifications eventually lead to failure of hUba1∼Ub thioester bond formation.

## 4 Methods

### 4.1 Extra-Precision (XP) Docking of Myricetin to hUba1 with Schrodinger’s Glide

The site-specific docking of Myr to human Uba1 (PDB ID: 6DC6) was carried out by forming a cubic grid (20 Å^3^) around the selected residues of each hotspot with the “Receptor Grid Generation” platform of Schrödinger’s Glide module. The structure (PDB ID: 6Dc6) with a resolution of 3.14 Å with *R*/*R*_free_ values of 0.214/0.251 was chosen for this study. The structure has been reported with missing coordinates for some amino acid residues which do not have significance on the overall structure, active site, or the binding site. This is the only human Uba1 crystal structure available to date with PDB ID: 6Dc6 and was chosen for this study as the previous reports on *in vivo* testing by this group were also done with the human Uba1. Myr and its analogues’ structures were downloaded in the .sdf format from the Pubchem database. All ligands were prepared for docking by 2D to three-dimensional (3D) molecular conversion with the LigPrep module using the default OPLS3e force field. All docking calculations were carried out with the XP protocol available in the Glide module. Even though Myr did not show strong docking scores at the HS-4 site and was not considered for XP docking, the analogues of Myr were docked at HS-4 as some of them showed high docking scores. The interaction plots of ligands with protein were generated using Ligplot+.^43^

### 4.2 Accelerated Molecular Dynamics (aMD) Simulations of the hUba1–Myr Complex

To understand the dynamics of ligand binding with Uba1, aMD was carried out for a total time of 300 ns. The ligand, Myr, was parameterized using *antechamber*, and the whole complex PDB file (hUba1 in complex with Myr) outputs from Schrödinger were stripped of all H atoms. The system was solvated with TIP3P water at a cutoff of 12.0, which added 47889 water residues to the system resulting in a cubic box of size of 120.65 × 122.75 × 124.00 Å and a volume of 1836550.27 Å^3^ for the complexes. The initial preparation of the protein–ligand complex for Amber simulation caused a renumbering of residues to 1–992 instead of 1–1057 (as in the PDB), as the program cannot account for missing residues in a structure for simulation. The solvated Uba1–Myr complex system was prepared for aMD in six consecutive steps, by a previously published operation.^44^ A Berendsen barostat and a Langevin thermostat were used for pressure and temperature scaling, respectively. SHAKE bond length constraints were applied to all bonds involving hydrogen. Short classical molecular dynamics run for 1 ns was also carried out before the aMD run to calculate the torsional and total energy boost parameters.

Following our previously published procedure,^44^ for each aMD simulation, particle mesh Ewald summation (PME) was used to calculate the electrostatic interactions. Long-range interactions were calculated with a cutoff of 10.0. The simulations were carried out at 300 K temperature and 2 fs time step. The National Information Infrastructure Development supercomputers of the University of Debrecen, Hungary, were sourced for running simulations on GPUs with the *pmemd.cuda* implementation of Amber14. The aMD simulations required extra parameters *E*_dihed_, α_dihed_, *E*_total_, and α_total_, which can be calculated using eq 1:
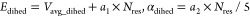
1
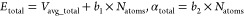
where *N*_res_ is the number of peptide residues (992 residues) and *N*_atoms_ is the total number of atoms in the system, which is 159,220 in the Uba1–Myr system. *V*_avg_dihed_ and *V*_avg_total_ are the average dihedral and total potential energies obtained from the classical MD run. The values of coefficients *a*_1_ and *a*_2_ were chosen to be 4 kcal/mol, and those of *b*_1_ and *b*_2_ were chosen to be 0.16 kcal/mol based on a previous study.^45^ The energy and boost information were saved at each 1000 time step. The Cartesian coordinate PCA was carried out using the *cpptraj* module.^46^*Grcarma*^46,47^ was used to generate the highest populated clusters using the top three PCs and write their representative structures in PDB format files.

### 4.3 Prediction of the Druggability Potential of Myr and Analogues

We used the SwissADME server to compute physicochemical descriptors as well as to predict the absorption, distribution, metabolism, and excretion (ADME) parameters, pharmacokinetic properties, drug-like nature, and medicinal chemistry friendliness of Myr and its analogues. On its webpage, the molecules to be studied were entered in their SMILES format.

## Supporting Information Available

Simulation of domain modulation within the hUba1 protein upon binding of Myr (MPG)
Radar plots calculated for selected compounds by the Swiss-ADME online server (PDF)

## Supplementary Material

ao3c03605_si_001.mpg

## References

[ref1] Goldknopf I. L.; Busch H. Isopeptide Linkage between Nonhistone and Histone-2a Polypeptides of Chromosomal Conjugate-Protein-A24. Proc. Natl. Acad. Sci. U.S.A. 1977, 74, 864–868. 10.1073/pnas.74.3.864. 265581 PMC430507

[ref2] Hunt L. T.; Dayhoff M. O. Amino-terminal sequence identity of ubiquitin and the nonhistone component of nuclear protein A24. Biochem. Biophys. Res. Commun. 1977, 74, 650–655. 10.1016/0006-291x(77)90352-7. 836318

[ref3] Varshavsky A. The early history of the ubiquitin field. Protein Sci. 2006, 15, 647–654. 10.1110/ps.052012306. 16501229 PMC2249785

[ref4] Hershko A. Ubiquitin: roles in protein modification and breakdown. Cell 1983, 34, 11–12. 10.1016/0092-8674(83)90131-9. 6309404

[ref5] Mansour M. A. Ubiquitination: Friend and foe in cancer. Int. J. Biochem. Cell Biol. 2018, 101, 80–93. 10.1016/j.biocel.2018.06.001. 29864543

[ref6] Walsh C. T.; Garneau-Tsodikova S.; Gatto G. J. Jr. Protein posttranslational modifications: the chemistry of proteome diversifications. Angew. Chem., Int. Ed. 2005, 44, 7342–7372. 10.1002/anie.200501023. 16267872

[ref7] Kelsall I. R. Non-lysine ubiquitylation: Doing things differently. Front. Mol. Biosci. 2022, 9, 1008175 10.3389/fmolb.2022.1008175. 36200073 PMC9527308

[ref8] Shmueli A.; Oren M. Life, death, and ubiquitin: taming the mule. Cell 2005, 121, 963–965. 10.1016/j.cell.2005.06.018. 15989944

[ref9] Suryadinata R.; Roesley S. N.; Yang G.; Sarcevic B. Mechanisms of generating polyubiquitin chains of different topology. Cells 2014, 3, 674–689. 10.3390/cells3030674. 24987835 PMC4197637

[ref10] Fenteany G.; Gaur P.; Sharma G.; Pinter L.; Kiss E.; Haracska L. Robust high-throughput assays to assess discrete steps in ubiquitination and related cascades. BMC Mol. Cell Biol. 2020, 21, 21 10.1186/s12860-020-00262-5. 32228444 PMC7106726

[ref11] Wilkinson K. D. Protein ubiquitination: a regulatory post-translational modification. Anticancer Drug Des 1987, 2, 211–229. 2835061

[ref12] Sun T.; Liu Z.; Yang Q. The role of ubiquitination and deubiquitination in cancer metabolism. Mol. Cancer 2020, 19, e1600200 10.1186/s12943-020-01262-x. PMC7529510 33004065

[ref13] Deng L.; Meng T.; Chen L.; Wei W.; Wang P. The role of ubiquitination in tumorigenesis and targeted drug discovery. Signal Transduction Targeted Ther. 2020, 5, 11 10.1038/s41392-020-0107-0. PMC7048745 32296023

[ref14] DeBerardinis R. J.; Chandel N. S. Fundamentals of cancer metabolism. Sci. Adv. 2016, 2, e1600200 10.1126/sciadv.1600200. 27386546 PMC4928883

[ref15] Fenteany G.; Gaur P.; Hegedus L.; Dudas K.; Kiss E.; Weber E.; Hackler L.; Martinek T.; Puskas L. G.; Haracska L. Multilevel structure-activity profiling reveals multiple green tea compound families that each modulate ubiquitin-activating enzyme and ubiquitination by a distinct mechanism. Sci. Rep. 2019, 9, 12801 10.1038/s41598-019-48888-6. 31488855 PMC6728334

[ref16] Gaur P. Discovery of Small-Molecule Inhibitors of Uba1 and Development of Step-Specific Assays for PCNA Ubiquitination. PhD Thesis, University of Szeged: Szeged, 2020. https://doktori.bibl.u-szeged.hu/id/eprint/10556/1/PhDThesis_Paras_Final.pdf.

[ref17] Fenteany G.; Sharma G.; Gaur P.; Borics A.; Weber E.; Kiss E.; Haracska L. A series of xanthenes inhibiting Rad6 function and Rad6-Rad18 interaction in the PCNA ubiquitination cascade. iScience 2022, 25, 104053 10.1016/j.isci.2022.104053. 35355521 PMC8958325

[ref18] Gaur P.; Fenteany G.; Tyagi C. Mode of inhibitory binding of epigallocatechin gallate to the ubiquitin-activating enzyme Uba1 via accelerated molecular dynamics. RSC Adv. 2021, 11, 8264–8276. 10.1039/d0ra09847g. 35423322 PMC8695214

[ref19] Sultana B.; Anwar F. Flavonols (kaempeferol, quercetin, myricetin) contents of selected fruits, vegetables and medicinal plants. Food Chem. 2008, 108, 879–884. 10.1016/j.foodchem.2007.11.053. 26065748

[ref20] Hou W.; Hu S.; Su Z.; Wang Q.; Meng G.; Guo T.; Zhang J.; Gao P. Myricetin attenuates LPS-induced inflammation in RAW 264.7 macrophages and mouse models. Future Med. Chem. 2018, 10, 2253–2264. 10.4155/fmc-2018-0172. 30095283

[ref21] Jiang M.; Zhu M.; Wang L.; Yu S. Anti-tumor effects and associated molecular mechanisms of myricetin. Biomed. Pharmacother. 2019, 120, 109506 10.1016/j.biopha.2019.109506. 31586904

[ref22] Stoll S.; Bitencourt S.; Laufer S.; Ines Goettert M. Myricetin inhibits panel of kinases implicated in tumorigenesis. Basic Clin. Pharmacol. Toxicol. 2019, 125, 3–7. 10.1111/bcpt.13201. 30624861

[ref23] Jiang S.; Tang X.; Chen M.; He J.; Su S.; Liu L.; He M.; Xue W. Design, synthesis and antibacterial activities against Xanthomonas oryzae pv. oryzae, Xanthomonas axonopodis pv. Citri and Ralstonia solanacearum of novel myricetin derivatives containing sulfonamide moiety. Pest Manage. Sci. 2020, 76, 853–860. 10.1002/ps.5587. 31419003

[ref24] Ortega J. T.; Suarez A. I.; Serrano M. L.; Baptista J.; Pujol F. H.; Rangel H. R. The role of the glycosyl moiety of myricetin derivatives in anti-HIV-1 activity in vitro. AIDS Res. Ther. 2017, 14, 57 10.1186/s12981-017-0183-6. 29025433 PMC5639754

[ref25] Hu T.; Yuan X.; Wei G.; Luo H.; Lee H. J.; Jin W. Myricetin-induced brown adipose tissue activation prevents obesity and insulin resistance in db/db mice. Eur. J. Nutr. 2018, 57, 391–403. 10.1007/s00394-017-1433-z. 28439667

[ref26] Chen M.; Chen Z.; Huang D.; Sun C.; Xie J.; Chen T.; Zhao X.; Huang J.; Li D.; Wu B.; et al. Corrigendum to “Myricetin inhibits TNF-alpha-induced inflammation in A549 cells via the SIRT1/NF-kappaB pathway”. Pulm. Pharmacol. Ther. 2021, 68, 102031 10.1016/j.pupt.2021.102031. 33863645

[ref27] Mondal S.; Jana J.; Sengupta P.; Jana S.; Chatterjee S. Myricetin arrests human telomeric G-quadruplex structure: a new mechanistic approach as an anticancer agent. Mol. Biosyst. 2016, 12, 2506–2518. 10.1039/c6mb00218h. 27249025

[ref28] Javed Z.; Khan K.; Herrera-Bravo J.; Naeem S.; Iqbal M. J.; Raza Q.; Sadia H.; Raza S.; Bhinder M.; Calina D.; et al. Myricetin: targeting signaling networks in cancer and its implication in chemotherapy. Cancer Cell Int. 2022, 22, 239 10.1186/s12935-022-02663-2. 35902860 PMC9336020

[ref29] Steinhart Z.; Angers S. Wnt signaling in development and tissue homeostasis. Development 2018, 145, dev146589 10.1242/dev.146589. 29884654

[ref30] Yao Y.; Lin G.; Xie Y.; Ma P.; Li G.; Meng Q.; Wu T. Preformulation studies of myricetin: a natural antioxidant flavonoid. Pharmazie 2014, 69, 19–26. 24601218

[ref31] Groen E. J. N.; Gillingwater T. H. UBA1: At the Crossroads of Ubiquitin Homeostasis and Neurodegeneration. Trends Mol. Med. 2015, 21, 622–632. 10.1016/j.molmed.2015.08.003. 26432019 PMC4596250

[ref32] Xu W.; Lukkarila J. L.; da Silva S. R.; Paiva S. L.; Gunning P. T.; Schimmer A. D. Targeting the ubiquitin E1 as a novel anti-cancer strategy. Curr. Pharm. Des. 2013, 19, 3201–3209. 10.2174/1381612811319180004. 23151135

[ref33] Volkamer A.; Griewel A.; Grombacher T.; Rarey M. Analyzing the topology of active sites: on the prediction of pockets and subpockets. J. Chem. Inf. Model. 2010, 50, 2041–2052. 10.1021/ci100241y. 20945875

[ref34] Hann Z. S.; Ji C.; Olsen S. K.; Lu X.; Lux M. C.; Tan D. S.; Lima C. D. Structural basis for adenylation and thioester bond formation in the ubiquitin E1. Proc. Natl. Acad. Sci. U.S.A. 2019, 116, 15475–15484. 10.1073/pnas.1905488116. 31235585 PMC6681703

[ref35] Lv Z.; Williams K. M.; Yuan L.; Atkison J. H.; Olsen S. K. Crystal structure of a human ubiquitin E1-ubiquitin complex reveals conserved functional elements essential for activity. J. Biol. Chem. 2018, 293, 18337–18352. 10.1074/jbc.RA118.003975. 30279270 PMC6254350

[ref36] Boer D. R.; Bijlmakers M. J. Differential Inhibition of Human and Trypanosome Ubiquitin E1S by TAK-243 Offers Possibilities for Parasite Selective Inhibitors. Sci. Rep. 2019, 9, 16195 10.1038/s41598-019-52618-3. 31700050 PMC6838199

[ref37] Hyer M. L.; Milhollen M. A.; Ciavarri J.; Fleming P.; Traore T.; Sappal D.; Huck J.; Shi J.; Gavin J.; Brownell J.; et al. A small-molecule inhibitor of the ubiquitin activating enzyme for cancer treatment. Nat. Med. 2018, 24, 186–193. 10.1038/nm.4474. 29334375

[ref38] Niu X.; Sun L.; Wang G.; Gao Y.; Yang Y.; Wang X.; Wang H. Investigation of the inhibition effect and mechanism of myricetin to Suilysin by molecular modeling. Sci. Rep. 2017, 7, 11748 10.1038/s41598-017-12168-y. 28924148 PMC5603505

[ref39] Song S.; Shao Z. From Myricetin to the Discovery of Novel Natural Human ENPP1 Inhibitors: A Virtual Screening, Molecular Docking, Molecular Dynamics Simulation, and MM/GBSA Study. Molecules 2022, 27, 6175 10.3390/molecules27196175. 36234712 PMC9573336

[ref40] Manach C.; Williamson G.; Morand C.; Scalbert A.; Remesy C. Bioavailability and bioefficacy of polyphenols in humans. I. Review of 97 bioavailability studies. Am. J. Clin. Nutr. 2005, 81, 230S–242S. 10.1093/ajcn/81.1.230S. 15640486

[ref41] Manach C.; Scalbert A.; Morand C.; Remesy C.; Jimenez L. Polyphenols: food sources and bioavailability. Am. J. Clin. Nutr. 2004, 79, 727–747. 10.1093/ajcn/79.5.727. 15113710

[ref42] Bhatt S.; Manhas D.; Kumar V.; Gour A.; Sharma K.; Dogra A.; Ojha P. K.; Nandi U. Effect of Myricetin on CYP2C8 Inhibition to Assess the Likelihood of Drug Interaction Using In Silico, In Vitro, and In Vivo Approaches. ACS Omega 2022, 7, 13260–13269. 10.1021/acsomega.2c00726. 35474783 PMC9026026

[ref43] Laskowski R. A.; Swindells M. B. LigPlot+: multiple ligand-protein interaction diagrams for drug discovery. J. Chem. Inf. Model. 2011, 51, 2778–2786. 10.1021/ci200227u. 21919503

[ref44] Tyagi C.; Marik T.; Szekeres A.; Vagvolgyi C.; Kredics L.; Otvos F. Tripleurin XIIc: Peptide Folding Dynamics in Aqueous and Hydrophobic Environment Mimic Using Accelerated Molecular Dynamics. Molecules 2019, 24, 358 10.3390/molecules24020358. 30669493 PMC6359335

[ref45] Pierce L. C.; Salomon-Ferrer R.; de Oliveira C. A. F.; McCammon J. A.; Walker R. C. Routine Access to Millisecond Time Scale Events with Accelerated Molecular Dynamics. J. Chem. Theory Comput. 2012, 8, 2997–3002. 10.1021/ct300284c. 22984356 PMC3438784

[ref46] Glykos N. M. Software news and updates. Carma: a molecular dynamics analysis program. J. Comput. Chem. 2006, 27, 1765–1768. 10.1002/jcc.20482. 16917862

[ref47] Koukos P. I.; Glykos N. M. Grcarma: A fully automated task-oriented interface for the analysis of molecular dynamics trajectories. J. Comput. Chem. 2013, 34, 2310–2312. 10.1002/jcc.23381. 24159629

