# Supplementary material for: Unraveling the Mechanism of Action of Myricetin in the Inhibition of hUba1∼Ubiquitin Thioester Bond Formation via In Silico Molecular Modeling Techniques
Source: ACS Omega. 2023 Aug 8;8(33):30432–41. doi: 10.1021/acsomega.3c03605 (PMC10448642; doi:10.1021/acsomega.3c03605)

Unraveling the mechanism of action of Myricetin in the inhibition of hUba1~ubiquitin thioester bond formation via *in silico* molecular modeling techniques

Paras Gaur[1], Chetna Tyagi[2*]

[1]Institute of Genetics, Biological Research Centre, Temesvári krt. 62, 6726 Szeged, Hungary

[2]Department of Microbiology, Faculty of Science and Informatics, University of Szeged, Közép fasor 52, H-6726 Szeged, Hungary

*Corresponding author

chetna.tyagi@bio.u-szeged.hu, cheta231@gmail.com

ORCID:

PG: 0000-0002-2112-389X

CT: 0000-0001-7067-4770

[1]**Current address:** Department of Biochemistry and Molecular Biology, Carver College of Medicine, University of Iowa, Iowa City, United States of America

Figure S1: The radar plots describing the ADME (absorption, distribution, metabolism, and excretion) properties of selected flavone compounds, namely, Myricetin, Fisetin, Isoquercetin, Kaempferol, Morin and Quercetin.

Myricetin

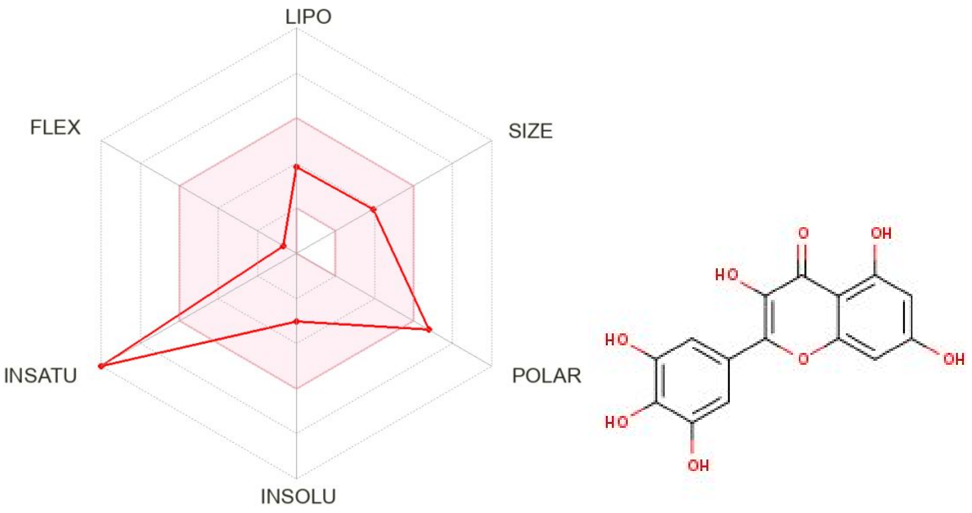

Fisetin

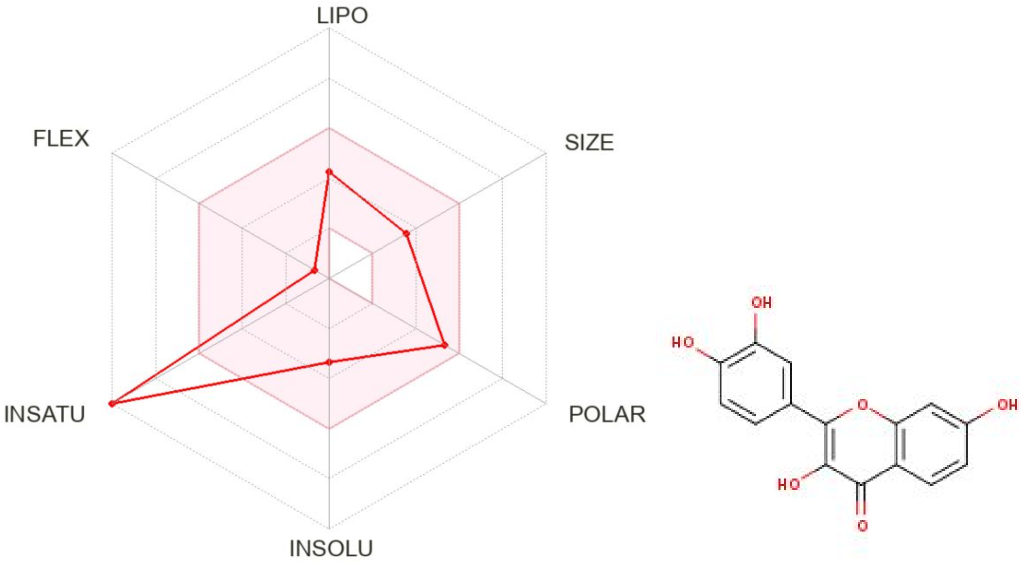

Isoquercetin

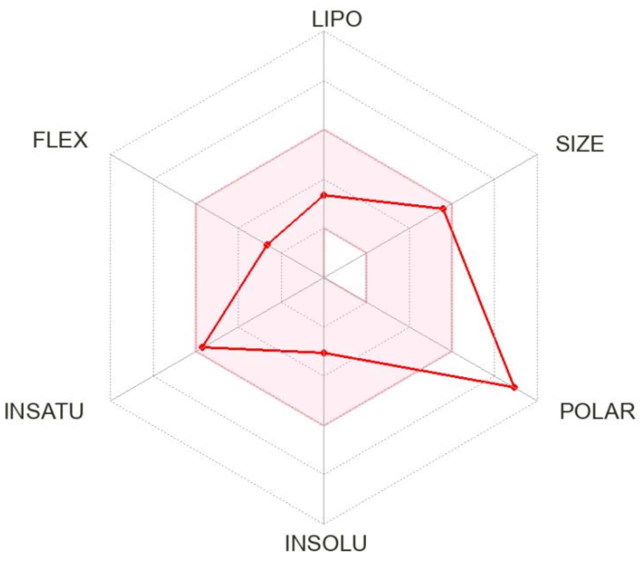

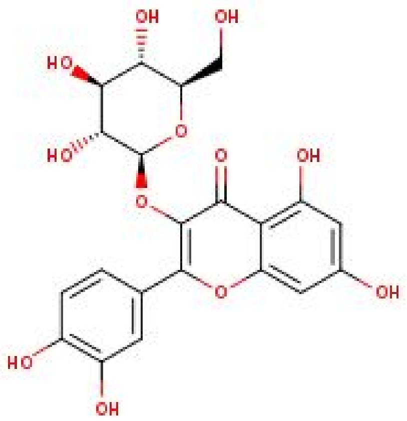

Kaempferol

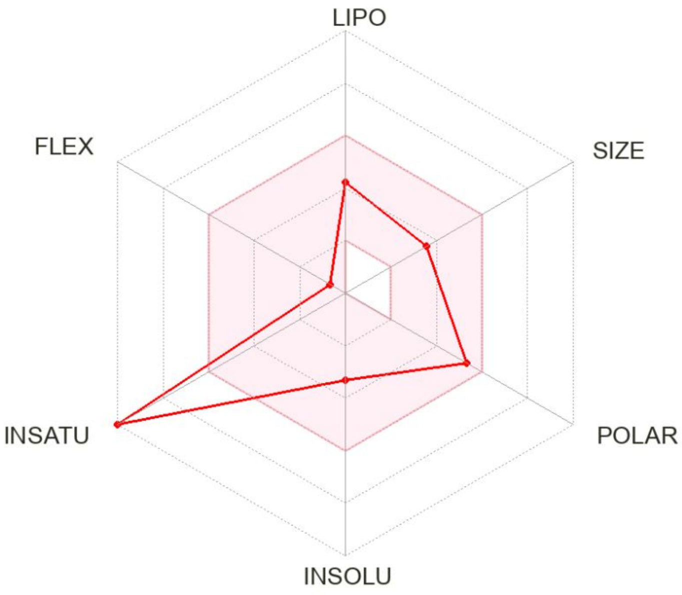

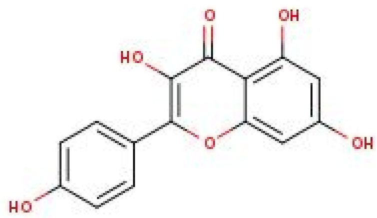

Morin

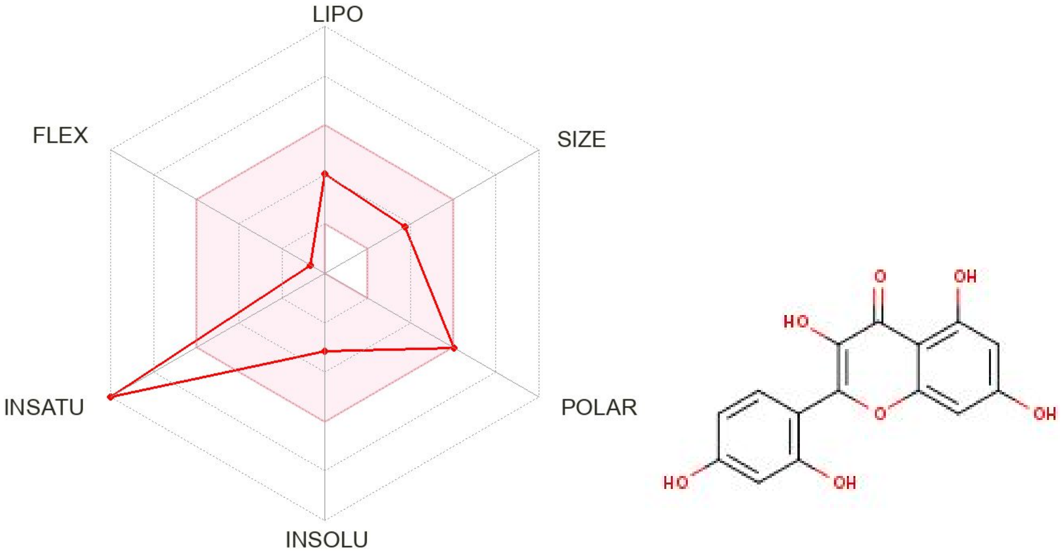

Quercetin

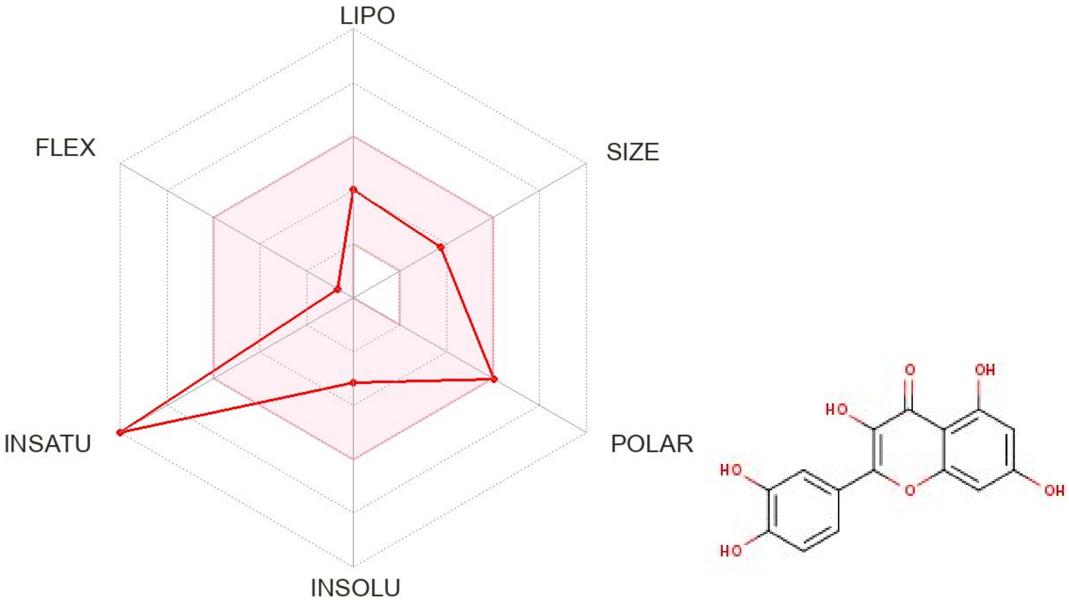

Supplement: Supplementary file 2 — ao3c03605_si_002.pdf [file ao3c03605_si_002.pdf]
